# Rethinking transport – towards clean and inclusive mobility (Highlights of the 2020 Transport Research Arena conference)

**DOI:** 10.1186/s12544-020-00434-6

**Published:** 2020-07-02

**Authors:** Miloš N. Mladenović, Pekka Leviäkangas, Claudio Roncoli, Saara Hänninen

**Affiliations:** 1 Aalto University, Espoo, Finland; 2 University of Oulu, Oulu, Finland; 3 VTT Technical Research Centre of Finland Ltd., Espoo, Finland

This topical collection includes contributions from Transport Research Arena (TRA) 2020, an international event that covers all transport modes and all aspects of mobility. The theme of the Transport Research Arena 2020 was “Rethinking transport – towards clean and inclusive mobility”. The theme itself highlights the complexity of transitioning towards sustainable mobility systems. At the centre of this transition is an urgent need to respond and adapt to the ongoing climate crisis. At the COP21 in 2015, European Union outlined the objective to reduce the greenhouse gas emissions by 40% below 1990 levels by 2030. In relation, mobility systems have a crucial role in achieving this target [1]. Beyond mitigation, we should urgently develop a range of adaptation mechanisms, as extreme weather phenomena will set new requirements for the whole system resilience, risk and disruption management, while changing energy consumption patterns. Even if these aspects of transition are complex enough in themselves to handle, our collective European values require a necessary trade-off by ensuring fairness during and from this transition, and delivering well-being and quality of life [2]. Taking into account the rich diversity of lifestyles and cultures across Europe, such transition means taking into account a multidimensional range of capabilities and needs that should be a basis for considering necessary changes in our everyday behaviour and norms.

This topical collection consists of 25 publications. One set of publications focuses on the development of improved models and algorithms using new data sources, highlighting the importance of new sensing technologies and their fusion, as well as opening access to existing databases. Kilpi et al. develop an algorithmic framework to extract relevant information about traffic dynamics from short-term traffic count data [3]. Calabrò et al. develop an algorithm for capacitated vehicle routing problem for inbound logistics using an ant-colony optimization approach [4]. Pretto et al. develop forecasting models for airport noise scenarios based on web data [5]. Finally, Diana et al. develop a model for understanding freight loading and unloading areas in the city based on GPS traces of logistics vehicles [6].

The second set of papers displays a range of aspects related to developing and evaluating emerging vehicle aspects, based on ongoing trends in digitalization, automation, and new energy sources that should be steered to support the ongoing transition. He et al. and Makridis et al.^1^ test the effects on traffic flow stability and energy efficiency from adaptive cruise control technology [7, 8]. Stevanovic & Mitrovic assess the traffic flow and safety impacts of alternate-directions lane assignment and reservation-based intersection control under the assumption of fully automated road vehicles [9]. Having in mind the development of electric powertrain for automated vehicles, Torabi et al. develop energy minimization algorithm for speed profile optimization [10]. In the maritime domain, Meersman et al. assess economic performance of vessel train as a semi-autonomous vessel platooning concept [11], while Arjona Aroca et al.^1^ assess the impact on fuel consumption and GHG emissions from the sea traffic management concept [12]. Finally, two papers take a close look at implications from new types of vehicles, one being high capacity freight vehicles [13]^1^ and another micro electric vehicles [14].

The third set of papers focuses on a multidimensional user perspective, providing the distributive analysis of impacts, as well as a range of insights from sociological and psychological angles. Fearnley & Aarhaug assess distributional impacts from subsidising urban and sub-urban public transport [15], while Malin et al.^1^ evaluate prevalence and characteristics of pedestrian fatalities and serious injuries in Finland [16]. In the context of users relation to emerging mobility technologies, Ramos et al.^1^ develop mobility profiles of both users and non-users of car sharing in several European cities [17], while both Matyas^1^ and Hoerler et al.^1^ provide an analysis of user needs, motivational and barrier factors for the use of mobility-as-a-service [18, 19]. Expanding the understanding of habit formation mechanisms, Karatsoli & Nathanail^1^ and Tiikkaja et al. highlight the importance of social media [20] and travel satisfaction [21] for mobility activity planning. Finally, focusing more on the user acceptance of new vehicle systems, Weber et al. studies the acceptance of attention-adaptive driver safety systems [22].

The final set of papers focuses more directly to diverse decision-making challenges that exist at various design, planning and policy levels in mobility systems. Starting from the roadway scale, Burghardt & Pashkevich focus on a challenge of selecting materials for horizontal road markings [23], while Budzynski et al. focus on assessing road restraint system functionality [24]. With a lens on asset management of large number of bridges, Allah Bukhsh et al.^1^ propose a framework for the development of optimal multi-year maintenance plans [25]. Highlighting the essential infrastructural challenge of large-scale failure, van der Tuin & Pel^1^ model impacts of passenger and freight disruptions [26]. Finally, Ellis provides a policy analysis of the European air market, taking into account geopolitical trends in and around Europe [27].

This diverse set of research efforts establishes a benchmark for future collaboration between TRA and ETRR in the coming years. We hope that this collaboration will further improve the level of research necessary for both technological (e.g., alternative energy sources, multimodal service integration) and institutional (e.g., intersectoral policy, networked governance, collaborative planning) rethinking of the transport sector. First, as ETRR follows the principles of open access, we hope that future research will advance further other aspects of open science framework, by providing access to data and algorithms, but also by opening scientific processes to a wider set of stakeholders and the public. Such efforts will be essential stepping-stones for putting into practice European initiatives on responsible research and innovation, and advancing the state-of-the-practice in action research. Second, we hope that future research activities will aim for expanding the diversity of its transdisciplinary approaches by further recognizing necessary knowledge from social science and humanities. Further rethinking of mobility systems will also have to rely on mixed methodologies, with a greater emphasis on complex systems and distributive justice theory. Finally, we sincerely hope that TRA will continue to be an essential place of learning and co-creation for a diverse set of actors across the civil society, sharing the responsibility for our common future.

## Footnotes

1 TRA2020 best paper recognition by the Scientific Panel
